# 2-Meth­oxy-6-[(*Z*)-[(5-methyl-2-pyrid­yl)imino­meth­yl]phenol

**DOI:** 10.1107/S1600536809031699

**Published:** 2009-08-15

**Authors:** Xin-Yu Liu, Yu-Hua Fan, Cai-Feng Bi, Qiang Wang, Yuan Gao

**Affiliations:** a Key Laboratory of Marine Chemistry Theory and Technology, Ministry of Education, College of Chemistry and Chemical Engineering, Ocean University of China, Qingdao, Shandong 266100, People’s Republic of China

## Abstract

The title compound, C_14_H_14_N_2_O_2_, was obtained by a condensation reaction between *o*-vanillin and 5-methyl­pyridin-2-amine. In the mol­ecule, the dihedral angle between the pyridine and benzene rings is 9.08 (13)°. An intra­molecular hydrogen bond involving the imine N atom and the hydroxyl group may influence the conformation of the mol­ecule. The crystal structure is stabilized by weak C—H⋯O hydrogen bonds.

## Related literature

For general background to the use of Schiff bases as ligands in coordination chemistry, see: Yamada, (1999 ▶). For their biological activity, see: Yang *et al.* (2000 ▶). For a related structure, see: Dal *et al.* (2007 ▶).
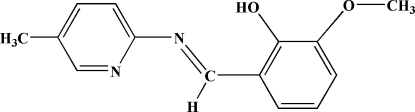


## Experimental

### 

#### Crystal data

C_14_H_14_N_2_O_2_
*M* *_r_* = 242.27
Monoclinic, 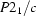

*a* = 11.5995 (6) Å
*b* = 4.9546 (2) Å
*c* = 23.9983 (12) Å
β = 117.6090 (4)°
*V* = 1222.15 (10) Å^3^
*Z* = 4
Mo *K*α radiation
μ = 0.09 mm^−1^
*T* = 296 K
0.42 × 0.10 × 0.10 mm

#### Data collection

Siemens SMART CCD area-detector diffractometer
Absorption correction: multi-scan (*SADABS*; Sheldrick, 1996 ▶) *T*_min_ = 0.963, *T*_max_ = 0.991
13275 measured reflections
2806 independent reflections
1822 reflections with *I* > 2σ(*I*)
*R*_int_ = 0.049

#### Refinement

*R*[*F* ^2^ > 2σ(*F* ^2^)] = 0.061
*wR*(*F* ^2^) = 0.198
*S* = 1.03
2806 reflections
166 parameters
H-atom parameters constrained
Δρ_max_ = 0.25 e Å^−3^
Δρ_min_ = −0.22 e Å^−3^

### 

Data collection: *SMART* (Siemens, 1996 ▶); cell refinement: *SAINT* (Siemens, 1996 ▶); data reduction: *SAINT*; program(s) used to solve structure: *SHELXS97* (Sheldrick, 2008 ▶); program(s) used to refine structure: *SHELXL97* (Sheldrick, 2008 ▶); molecular graphics: *SHELXTL* (Sheldrick, 2008 ▶); software used to prepare material for publication: *SHELXTL*.

## Supplementary Material

Crystal structure: contains datablocks I, global. DOI: 10.1107/S1600536809031699/lh2873sup1.cif

Structure factors: contains datablocks I. DOI: 10.1107/S1600536809031699/lh2873Isup2.hkl

Additional supplementary materials:  crystallographic information; 3D view; checkCIF report

## Figures and Tables

**Table 1:** Hydrogen-bond geometry (Å, °)

*D*—H⋯*A*	*D*—H	H⋯*A*	*D*⋯*A*	*D*—H⋯*A*
O1—H1⋯N2	0.82	1.84	2.5587 (19)	146
C3—H3⋯O2^i^	0.93	2.64	3.567 (3)	175
C4—H4⋯O1^i^	0.93	2.66	3.282 (2)	125

Symmetry code: (i) 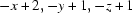.

**Fig. 1.**
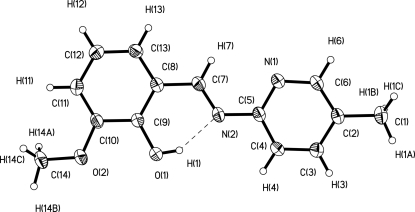
The molecular structure of the title compound, showing 30% probability displacement ellipsoids and the atom-numbering scheme. The dashed line indicates a hydrogen bond.

**Fig. 2.**
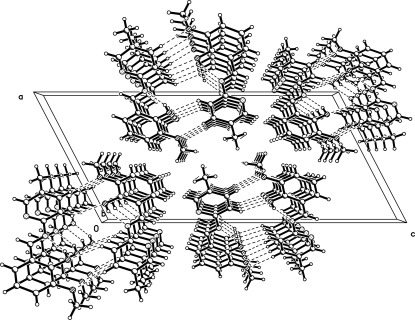
Part of the crystal structure with hydrogen bonds shown as dashed lines.

## supplementary crystallographic information

### Comment

Schiff bases are used extensively as ligands in the field of coordination chemistry (Yamada, 1999), and thay have diverse biological activities, such as antibacterial and antitumor activities (Yang *et al.*, 2000). An example of the crystal structure of one such compound, 2-[(1E)-2-aza-2-(5-methyl(2-pyridyl)ethenyl)]-4-bromobenzen-1-ol, is available in the literature (Dal *et al.*, 2007). Herein, the crystal structure of the title compound is presented.

The molecular structure of the title compound is shown in Fig. 1. The molecule contains two aromatic rings linked through a imine group. the dihedral angle between the pyridine and the benzene ring is 9.08 (13)°. An intramolecular O—H···N hydrogen bond in the molecular structure is similar to that in the reported structure (Dal *et al.*, 2007). The crystal structure is stabilized by very weak C-H···O hydrogen bonds (Fig. 2).

### Experimental

All chemicals were analytical reagent grade and used directly without further purification. O-vanillin (1 mmol, 152.1 mg) was added with stirring to anhydrous ethanol (30 ml) and the mixture was slowly dropped into an anhydrous ethanol solution (15 ml) containing (1 mmol, 108.1 mg) 5-methylpyridin-2-amine at 339 K and was then stirred for 4 h, a red solid then separated out. The product was collected by filtration and washed several times with anhydrous ethanol and dried under vacuum. Red single crystals suitable for X-ray diffraction were obtained after 4 days by slow evaporation at room temperature of an anhydrous ethanol solution of the title compound.

### Refinement

All H-atoms were positioned geometrically and refined using a riding model, with C—H = 0.93 Å or 0.96 Å (methyl) and  0.82 Å (hydroxyl) and *U*_iso_(H) =1.2*U*_eq_(C) or 1.2*U*_eq_(C_methyl_ and O).

### Crystal data

**Table:**

C_14_H_14_N_2_O_2_	*F*(000) = 512
*M**_r_* = 242.27	*D*_x_ = 1.317 Mg m^−^^3^
Monoclinic, *P*2_1_/*c*	Mo *K*α radiation, λ = 0.71073 Å
Hall symbol: -P 2ybc	Cell parameters from 2267 reflections
*a* = 11.5995 (6) Å	θ = 3.3–24.6°
*b* = 4.9546 (2) Å	µ = 0.09 mm^−^^1^
*c* = 23.9983 (12) Å	*T* = 296 K
β = 117.6090°	Neddle, red
*V* = 1222.15 (10) Å^3^	0.42 × 0.10 × 0.10 mm
*Z* = 4	

### Data collection

**Table:**

Siemens SMART CCD area-detector diffractometer	2806 independent reflections
Radiation source: fine-focus sealed tube	1822 reflections with *I* > 2σ(*I*)
graphite	*R*_int_ = 0.049
φ and ω scans	θ_max_ = 27.5°, θ_min_ = 1.9°
Absorption correction: multi-scan (*SADABS*; Sheldrick, 1996)	*h* = −14→15
*T*_min_ = 0.963, *T*_max_ = 0.991	*k* = −6→6
13275 measured reflections	*l* = −31→30

### Refinement

**Table:**

Refinement on *F*^2^	Primary atom site location: structure-invariant direct methods
Least-squares matrix: full	Secondary atom site location: difference Fourier map
*R*[*F*^2^ > 2σ(*F*^2^)] = 0.061	Hydrogen site location: inferred from neighbouring sites
*wR*(*F*^2^) = 0.198	H-atom parameters constrained
*S* = 1.03	*w* = 1/[σ^2^(*F*_o_^2^) + (0.1232*P*)^2^ + 0.0489*P*] where *P* = (*F*_o_^2^ + 2*F*_c_^2^)/3
2806 reflections	(Δ/σ)_max_ < 0.001
166 parameters	Δρ_max_ = 0.25 e Å^−^^3^
0 restraints	Δρ_min_ = −0.22 e Å^−^^3^

### Special details

**Table:**

Geometry. All e.s.d.'s (except the e.s.d. in the dihedral angle between two l.s. planes) are estimated using the full covariance matrix. The cell e.s.d.'s are taken into account individually in the estimation of e.s.d.'s in distances, angles and torsion angles; correlations between e.s.d.'s in cell parameters are only used when they are defined by crystal symmetry. An approximate (isotropic) treatment of cell e.s.d.'s is used for estimating e.s.d.'s involving l.s. planes.
Refinement. Refinement of *F*^2^ against ALL reflections. The weighted *R*-factor *wR* and goodness of fit *S* are based on *F*^2^, conventional *R*-factors *R* are based on *F*, with *F* set to zero for negative *F*^2^. The threshold expression of *F*^2^ > σ(*F*^2^) is used only for calculating *R*-factors(gt) *etc*. and is not relevant to the choice of reflections for refinement. *R*-factors based on *F*^2^ are statistically about twice as large as those based on *F*, and *R*- factors based on ALL data will be even larger.

### Fractional atomic coordinates and isotropic or equivalent isotropic displacement parameters (Å^2^)

**Table:**

	*x*	*y*	*z*	*U*_iso_*/*U*_eq_	
C1	1.3545 (2)	1.1391 (4)	0.42144 (10)	0.0552 (6)	
H1A	1.4361	1.0529	0.4317	0.083*	
H1B	1.3646	1.2560	0.4553	0.083*	
H1C	1.3275	1.2431	0.3837	0.083*	
C2	1.25384 (18)	0.9287 (4)	0.41151 (9)	0.0435 (5)	
C3	1.2184 (2)	0.8629 (4)	0.45776 (9)	0.0518 (5)	
H3	1.2577	0.9487	0.4966	0.062*	
C4	1.1247 (2)	0.6699 (4)	0.44540 (10)	0.0521 (5)	
H4	1.1000	0.6241	0.4759	0.063*	
C5	1.06743 (18)	0.5443 (4)	0.38751 (9)	0.0418 (5)	
C6	1.18963 (19)	0.7932 (4)	0.35514 (9)	0.0470 (5)	
H6	1.2118	0.8371	0.3236	0.056*	
C7	0.92176 (17)	0.2020 (4)	0.32757 (8)	0.0411 (5)	
H7	0.9509	0.2221	0.2976	0.049*	
C8	0.82147 (17)	0.0051 (4)	0.31741 (8)	0.0388 (4)	
C9	0.77601 (18)	−0.0253 (4)	0.36199 (9)	0.0420 (5)	
C10	0.67807 (19)	−0.2169 (4)	0.35134 (9)	0.0454 (5)	
C11	0.62864 (19)	−0.3719 (4)	0.29787 (9)	0.0473 (5)	
H11	0.5642	−0.4986	0.2910	0.057*	
C12	0.6744 (2)	−0.3404 (4)	0.25398 (9)	0.0477 (5)	
H12	0.6396	−0.4453	0.2177	0.057*	
C13	0.76972 (19)	−0.1575 (4)	0.26345 (9)	0.0444 (5)	
H13	0.8004	−0.1407	0.2340	0.053*	
C14	0.5556 (2)	−0.4409 (5)	0.39416 (11)	0.0647 (7)	
H14A	0.4731	−0.4166	0.3576	0.097*	
H14B	0.5437	−0.4396	0.4312	0.097*	
H14C	0.5926	−0.6105	0.3913	0.097*	
N1	1.09794 (16)	0.6034 (3)	0.34181 (7)	0.0468 (4)	
N2	0.97078 (15)	0.3485 (3)	0.37709 (7)	0.0439 (4)	
O1	0.82133 (16)	0.1211 (3)	0.41479 (7)	0.0617 (5)	
H1	0.8722	0.2352	0.4145	0.093*	
O2	0.64070 (16)	−0.2284 (3)	0.39775 (7)	0.0662 (5)	

### Atomic displacement parameters (Å^2^)

**Table:**

	*U*^11^	*U*^22^	*U*^33^	*U*^12^	*U*^13^	*U*^23^
C1	0.0492 (12)	0.0508 (13)	0.0651 (13)	−0.0111 (10)	0.0261 (10)	0.0039 (10)
C2	0.0383 (10)	0.0425 (11)	0.0525 (11)	−0.0024 (8)	0.0235 (8)	0.0047 (8)
C3	0.0529 (12)	0.0577 (13)	0.0463 (11)	−0.0142 (10)	0.0241 (9)	−0.0060 (9)
C4	0.0572 (13)	0.0622 (13)	0.0474 (11)	−0.0159 (10)	0.0330 (10)	−0.0029 (9)
C5	0.0396 (10)	0.0420 (11)	0.0496 (10)	−0.0038 (8)	0.0255 (9)	0.0022 (8)
C6	0.0483 (11)	0.0510 (12)	0.0521 (11)	−0.0084 (9)	0.0320 (9)	0.0024 (9)
C7	0.0359 (9)	0.0466 (11)	0.0447 (10)	0.0008 (8)	0.0220 (8)	0.0051 (8)
C8	0.0368 (10)	0.0375 (10)	0.0445 (10)	0.0010 (7)	0.0209 (8)	0.0034 (7)
C9	0.0426 (10)	0.0428 (11)	0.0457 (10)	−0.0078 (8)	0.0248 (8)	−0.0038 (8)
C10	0.0443 (10)	0.0465 (11)	0.0553 (11)	−0.0076 (8)	0.0314 (9)	−0.0038 (8)
C11	0.0422 (10)	0.0428 (11)	0.0562 (11)	−0.0065 (8)	0.0222 (9)	−0.0024 (8)
C12	0.0497 (11)	0.0462 (11)	0.0452 (10)	−0.0023 (9)	0.0203 (9)	−0.0047 (8)
C13	0.0465 (11)	0.0476 (12)	0.0445 (10)	−0.0003 (8)	0.0255 (9)	0.0004 (8)
C14	0.0669 (15)	0.0673 (15)	0.0784 (16)	−0.0255 (12)	0.0493 (13)	−0.0062 (12)
N1	0.0469 (9)	0.0522 (10)	0.0478 (9)	−0.0090 (8)	0.0274 (8)	−0.0010 (7)
N2	0.0397 (9)	0.0464 (10)	0.0512 (9)	−0.0083 (7)	0.0257 (7)	0.0000 (7)
O1	0.0707 (10)	0.0734 (11)	0.0594 (9)	−0.0352 (8)	0.0457 (8)	−0.0245 (8)
O2	0.0782 (11)	0.0697 (11)	0.0738 (10)	−0.0374 (9)	0.0548 (9)	−0.0209 (8)

### Geometric parameters (Å, °)

**Table:**

C1—C2	1.500 (3)	C8—C13	1.402 (3)
C1—H1A	0.9600	C8—C9	1.403 (2)
C1—H1B	0.9600	C9—O1	1.338 (2)
C1—H1C	0.9600	C9—C10	1.410 (3)
C2—C6	1.379 (3)	C10—O2	1.371 (2)
C2—C3	1.389 (3)	C10—C11	1.372 (3)
C3—C4	1.373 (3)	C11—C12	1.391 (3)
C3—H3	0.9300	C11—H11	0.9300
C4—C5	1.380 (3)	C12—C13	1.365 (3)
C4—H4	0.9300	C12—H12	0.9300
C5—N1	1.332 (2)	C13—H13	0.9300
C5—N2	1.414 (2)	C14—O2	1.418 (2)
C6—N1	1.342 (2)	C14—H14A	0.9600
C6—H6	0.9300	C14—H14B	0.9600
C7—N2	1.279 (2)	C14—H14C	0.9600
C7—C8	1.450 (3)	O1—H1	0.8200
C7—H7	0.9300		
			
C2—C1—H1A	109.5	C9—C8—C7	120.01 (16)
C2—C1—H1B	109.5	O1—C9—C8	122.83 (17)
H1A—C1—H1B	109.5	O1—C9—C10	117.82 (16)
C2—C1—H1C	109.5	C8—C9—C10	119.36 (17)
H1A—C1—H1C	109.5	O2—C10—C11	125.75 (17)
H1B—C1—H1C	109.5	O2—C10—C9	114.34 (17)
C6—C2—C3	116.38 (17)	C11—C10—C9	119.91 (18)
C6—C2—C1	121.39 (17)	C10—C11—C12	120.31 (18)
C3—C2—C1	122.22 (18)	C10—C11—H11	119.8
C4—C3—C2	119.25 (18)	C12—C11—H11	119.8
C4—C3—H3	120.4	C13—C12—C11	120.83 (18)
C2—C3—H3	120.4	C13—C12—H12	119.6
C3—C4—C5	119.68 (18)	C11—C12—H12	119.6
C3—C4—H4	120.2	C12—C13—C8	120.16 (17)
C5—C4—H4	120.2	C12—C13—H13	119.9
N1—C5—C4	122.84 (17)	C8—C13—H13	119.9
N1—C5—N2	119.77 (17)	O2—C14—H14A	109.5
C4—C5—N2	117.37 (17)	O2—C14—H14B	109.5
N1—C6—C2	125.65 (17)	H14A—C14—H14B	109.5
N1—C6—H6	117.2	O2—C14—H14C	109.5
C2—C6—H6	117.2	H14A—C14—H14C	109.5
N2—C7—C8	120.97 (17)	H14B—C14—H14C	109.5
N2—C7—H7	119.5	C5—N1—C6	116.19 (16)
C8—C7—H7	119.5	C7—N2—C5	121.88 (16)
C13—C8—C9	119.41 (17)	C9—O1—H1	109.5
C13—C8—C7	120.57 (17)	C10—O2—C14	117.02 (16)

### Hydrogen-bond geometry (Å, °)

**Table:**

*D*—H···*A*	*D*—H	H···*A*	*D*···*A*	*D*—H···*A*
O1—H1···N2	0.82	1.84	2.5587 (19)	146
C3—H3···O2^i^	0.93	2.64	3.567 (3)	175
C4—H4···O1^i^	0.93	2.66	3.282 (2)	125

Symmetry codes: (i) −*x*+2, −*y*+1, −*z*+1.
